# PROSPECTIVE MULTI-OMIC ANALYSIS OF HUMAN LONGEVITY COHORTS IDENTIFIES ANALYTE NETWORKS ASSOCIATED WITH LONGEVITY

**DOI:** 10.1093/geroni/igad104.2245

**Published:** 2023-12-21

**Authors:** Lance Pflieger, Kengo Watanabe, Max Robinson, Gustavo Glusman, Jodi Lapidus, Oliver Fiehn, Robert Moritz, Noa Rappaport

**Affiliations:** Phenome Health, Seattle, Washington, United States; Institute for Systems Biology, Seattle, Washington, United States; Institute for Systems Biology, Seattle, Washington, United States; Institute for Systems Biology, Seattle, Washington, United States; Oregon Health & Science University, Portland, Oregon, United States; UC Davis, Davis, California, United States; Institute for Systems Biology, Seattle, Washington, United States; Institute for Systems Biology, Seattle, Washington, United States

## Abstract

Serum based biomarkers of longevity have long been sought to explain the mechanisms of healthy aging and longevity. Using a 1:3 case cohort design, the Longevity Consortium has produced untargeted mass spectrometry based proteomic and metabolomic datasets from serum of four cohorts with longevity status, defined as those that reached the age corresponding to the 98th percentile of survival using sex specific and birth cohort specific survival percentiles. The cohorts are the Osteoporotic Fractures in Men study, the Study of Osteoporotic Fractures, the Health, Aging, and Body Composition Study, and the Cardiovascular Health Study. In this study, we integrate metabolomics and proteomics using machine learning and system biology approaches to construct multi-omic signatures predictive of longevity and healthy aging. We identify networks enriched for biomarkers previously shown to be associated with longevity such as apolipoproteins, along with novel associations, and we further compare with our findings in a mouse omics LC dataset of molecular changes induced by life-extending interventions. We show substantial differences between male and female longevity networks. The study highlights the effectiveness of using integrative systems biology methods to capture the heterogeneity of underlying molecular aging phenotypes, in order to generate a robust signature of longevity. The identified biomarker signatures may have significant implications for the development of personalized interventions aimed at promoting healthy aging and preventing age-related diseases.

